# Experimental study on the inhibitory effect of bolting position on crack propagation in cavity-containing sandstone

**DOI:** 10.1371/journal.pone.0344102

**Published:** 2026-02-27

**Authors:** Dong Zhu, Yi-jiang Zong, Xiao-fei Liu, Bin Du, Shu-xue Ding

**Affiliations:** 1 School of Transportation Engineering, Jiangsu Vocational Institute of Architectural Technology, Xuzhou Jiangsu Province, China; 2 Jiangsu Collaborative Innovation Center for Green Smart Cities and Heritage Conservation, Xuzhou Jiangsu Province, China; 3 School of Safety Engineering, China University of Mining and Technology, Xuzhou, Jiangsu Province, China; 4 School of Civil Engineering, Henan Polytechnic University, Jiaozuo, Henan Province, China; Islamic Azad University Mashhad Branch, IRAN, ISLAMIC REPUBLIC OF

## Abstract

This study systematically investigated the effects of seven bolt anchorage positions on the mechanical behavior and failure of single-hole sandstone specimens using uniaxial compression tests and PFC^2D^ simulations, with emphasis on crack-propagation suppression. The results showed that: (1) Anchorage position strongly influenced the stress-strain response, mechanical parameters, crack initiation, and final failure morphology. When the bolt passed through the cavity center (1#) or was tangent to the cavity crown (2#), both strength and stiffness decreased, the pre-peak response showed multiple stress drops, and post-peak failure remained brittle. By contrast, anchoring 12–18 mm from the cavity center (3#–4#) increased strength and *E*, and the post-peak response shifted from brittle to more ductile (plastic-like) behavior. Position 3# yielded the best overall performance, with peak strength, *E*, secant modulus, and crack initiation stress increasing by 42.72%, 44.61%, 71.93%, and 44.39%, respectively. (2) Failure patterns depended on bolt location: near-cavity anchoring promoted stress concentration and a V-shaped crown collapse; intermediate anchoring (3#–5#) produced a distinct partitioned failure between upper and lower regions; more distant anchoring (6#–7#) increased damage severity and led to more complex fracture morphologies. (3) Simulations indicated that position 3# achieved the highest energy storage capacity (*K*_*max*_ = 0.91) and the greatest resistance to instability, whereas position 2# showed the lowest capacity (*K*_*min*_= 0.29) and the highest failure susceptibility. Positions 4#–5# formed a favorable range in which higher bolt axial forces generated a localized displacement field that inhibited crack growth and confined the damage zone. These findings show that selecting an appropriate anchorage position can markedly improve stiffness, load capacity, and crack resistance of cavity-containing sandstone, providing quantitative guidance for bolt layout in engineering practice.

## Introduction

Sandstone is a common sedimentary rock in geotechnical engineering, and its mechanical behavior is a key determinant of structural stability. However, sandstone frequently contains internal defects formed by geological processes and environmental weathering [1–3], among which cavities are particularly prevalent. As internal discontinuities, cavities can markedly degrade the mechanical performance of sandstone across multiple metrics [4–7].

In recent years, increasing attention has been directed toward the mechanical response and failure mechanisms of rocks containing cavities. These issues have been investigated largely through laboratory experiments and numerical simulations. Experimentally, Wu et al. [8–10] conducted uniaxial compression tests on brittle sandstone with different cavity geometries to quantify key mechanical parameters (e.g., compressive strength, *E*, and peak strain) and to characterize failure and damage evolution. Using acoustic emission (AE) monitoring, Li et al. [11] demonstrated that cavity size can substantially alter the failure modes of rock-like materials. Under biaxial loading, Liao et al. [12] clarified crack propagation patterns around a single hole and the associated local strain fields in rock-like specimens. Ji et al. [13] combined localized compression loading with digital image correlation (DIC), infrared thermography, and high-speed imaging to capture progressive instability and failure mechanisms in centrally holed specimens under non-uniform loading. Huang et al. [14] integrated optical imaging and AE monitoring to document real-time crack propagation and AE signatures in sandstone containing an elliptical cavity. Li et al. [15] systematically assessed how cavity geometry (size, shape, and inclination) influences marble strength and fracture behavior, and concluded that tensile cracking is controlled by strain localization.

Numerically, the discrete element method (DEM), including PFC^2D^, has been widely adopted to model cavity-induced damage processes. Using simulated uniaxial compression, Lotidis [16] and Wong [17] reported that both the size and position of circular holes strongly affect strength and elastic modulus, and suggested that crack initiation is closely associated with the release and redistribution of stress concentrations. Zhang [18], Chen [19], and Xia et al. [20] further examined how cavity type governs stress distribution, mechanical response, and failure patterns. Combining laboratory experiments and PFC^2D^ simulations, Yao [21] evaluated how defect size and spatial location (including both holes and pores) affect mechanical properties, crack growth, and stress evolution. Fan [22] developed combined hole-pore models to investigate how fissures modulate damage evolution in cavity-containing specimens. Yang et al. [23] used simulations to quantify the effects of lateral pressure coefficient, joint dip angle, and rock-bridge length on failure modes and crack evolution around circular defects. Wang et al. [24] systematically examined hole-fissure interaction by varying fissure dip angle, pore spacing, and spatial configuration, and assessed their impacts on mechanical properties and fracture processes.

Collectively, prior work has emphasized how cavity geometry, loading conditions, and interactions among multiple defects influence rock mechanical properties and failure modes. These experimental and numerical approaches have substantially advanced understanding of damage accumulation and failure mechanisms in cavity-bearing rock.

Rock bolts are widely used for reinforcement in tunneling, hydraulic engineering, and mining, and are particularly effective for stabilizing fractured or otherwise unstable rock masses. Their effectiveness depends on a clear understanding of anchorage mechanisms. However, bolting involves complex load-transfer and interaction processes, which have been studied extensively. In defective rock masses in particular, many studies have investigated factors that control anchorage performance. Reported factors include rock type [25–27], bolt material and mechanical properties, joint roughness [28,29], and bolt inclination [30]. Additional work has characterized the mechanical response, failure modes [31–33], flexural behavior [34,35], and shear performance [36–38] of bolted rock masses. Nevertheless, the crack-arresting mechanisms of rock bolts and their control of crack propagation under loading remain insufficiently understood and require further investigation.

Existing studies on bolting in defective rock masses often rely on rock-like analog materials with prefabricated flaws to evaluate anchorage behavior [39–42]. In contrast, anchorage mechanisms in sandstone containing localized cavities, a frequent condition in practice, have received comparatively limited attention. Specifically, how the relative bolt-cavity position influences surrounding-rock stability remains unclear. Accordingly, clarifying the effects of bolt location on crack propagation and failure patterns in cavity-containing rock under constant pretension is of clear engineering relevance.

Here, we combine laboratory experiments with discrete element simulations to systematically quantify how anchorage position regulates the mechanical response and failure modes of cavity-containing sandstone. We identify an optimal anchorage range relative to the cavity center, moving beyond the qualitative notion of an “optimal position” commonly used in prior analyses. Simulations further elucidate the underlying mechanisms, including crack suppression associated with higher bolt axial force, increased elastic energy conversion, and the formation of a localized displacement field. These findings provide quantitative guidance and a mechanistic basis for precision anchorage design in defective rock masses.

## Sample preparation and loading

### Sample preparation

Red sandstone was collected from Zigong, Sichuan Province, China. X-ray diffraction (*XRD*) showed that the sandstone was mainly composed of quartz, feldspar, and calcite, with minor hematite, mica, montmorillonite, and kaolinite. T The rock had a medium-grained texture with relatively uniform particles and a dense fabric, with an average density of approximately 2.48 × 10³ kg/m³.

Following established procedures [43–45], intact blocks were cut, ground, and machined into rectangular specimens (80 mm width × 160 mm height × 40 mm thickness). This geometry enabled clear observation of crack initiation at the cavity, subsequent propagation, and eventual instability. The 2:1 height-to-width ratio provided an adequate free path for crack growth while reducing end-boundary effects, improving capture of the evolving fracture process. In addition, this aspect ratio better approximates the classical “semi-infinite body with a hole” assumption, supporting subsequent theoretical interpretation and numerical validation of crack-propagation mechanisms.

**Fig 1:**
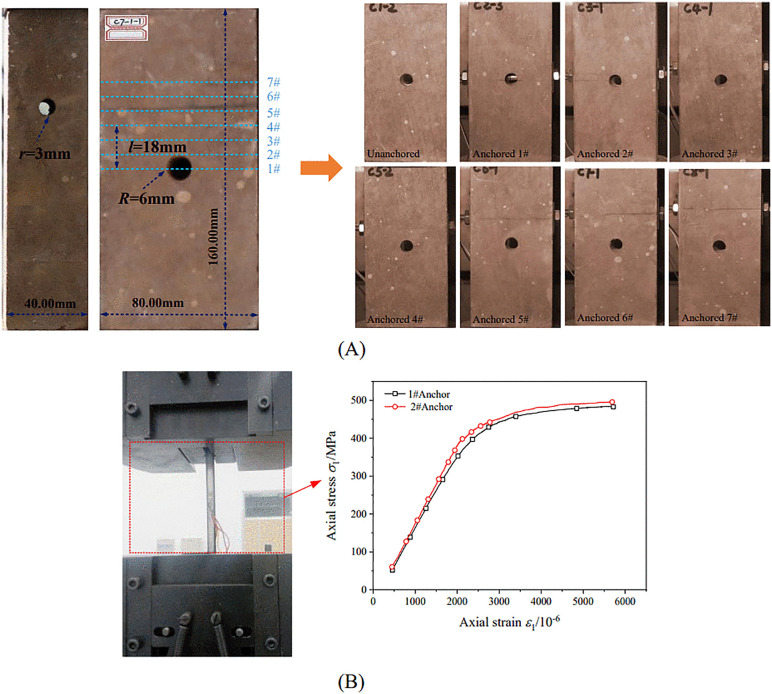
Specimen preparation and bolt mechanical testing. (A) red sandstone specimen with a single hole and bolt anchorage configuration; (B) bolt pull-out test and its corresponding stress-strain curve.

Each specimen was prefabricated with one circular cavity (radius *R* = 6 mm) and a bolt borehole (radius *r* = 3 mm), as shown in Fig 1A. The center-to-center distance between the bolt hole and the cavity was defined as *l*. To account for potential end effects, seven *l* values were considered: 0, 6, 12, 18, 24, 30, and 36 mm.

Steel rods (6 mm diameter) were selected to represent bolts commonly used in geotechnical applications. The rods had an elastic modulus of approximately 196.65 GPa, a tensile strength of about 489.25 MPa, and a unit weight of approximately 27 kN/m³. The corresponding stress-strain curve is provided in Fig 1B. Steel plates and nuts were used as anchorage components. Pretension was applied via nuts and plates at both ends to simulate the end-anchored bolting method commonly used in practice. To address scale differences between laboratory specimens and in-situ rock masses, a laboratory-to-field torque conversion was derived using similarity theory [46]. The torque similarity ratio *C*_*T*_ is defined as:


$$C_{T}=T_{p}/T_{m}=(K_{p}\cdot F_{p}\cdot d_{p})/(K_{m}\cdot F_{m}\cdot d_{m})$$ (1)

Rewriting Eq 1 yields:


$$C_{T}=(K_{p}/K_{m})\cdot (d_{p}/d_{m})\cdot (F_{p}/F_{m})$$ (2)

The ratio *K*_*p*_/*K*_*m*_ is defined as the similarity ratio of the torque coefficient, denoted as *C*_*K*_. The ratio *d*_*p*_/*d*_*m*_ is defined as the similarity ratio of diameter, expressed as *C*_*d*_, where *C*_*d*_ = *C*_*L*_, and *C*_*L*_ represents the geometric similarity ratio of the bolt. The ratio *F*_*p*_/*F*_*m*_ is defined as the similarity ratio of axial force, denoted as *C*_*F*_, where:


$$C_{F}=C_{\gamma }\cdot {\left(C_{L}\right)}^{\text{3}}$$ (3)

*C*_*γ*_ is defined as the similarity ratio of the unit weight of the bolt.

Substituting *C*_*K*_, *C*_*F*_ and *C*_*d*_ into Eq 2 gives the torque similarity ratio for bolts as:


$$C_{T}=C_{K}\cdot C_{F}\cdot C_{d}=\;C_{K}\cdot C_{\gamma }\cdot {\left(C_{L}\right)}^{\text{3}}\cdot C_{L}=\;C_{K}\cdot C_{\gamma }\cdot {\left(C_{L}\right)}^{\text{4}}$$ (4)

According to similarity theory, complete similarity of the physical processes between the model and the prototype requires that all corresponding dimensionless parameters remain identical in both systems. Therefore, the condition *K*_*m*_ = *K*_*p*_ must be satisfied, i.e., the torque coefficient of the model bolt equals that of the prototype bolt, and thus *C*_*K*_ = 1. The torque scaling ratio *C*_*T*_ was calculated as as: *C*_*T*_ = 1× (78.5/27) × (25/6)^4^ = 876.32.

All experiments were conducted at the State Key Laboratory for Geomechanics and Deep Underground Engineering, China University of Mining and Technology. In the field, high-strength rock bolts have a diameter of approximately 25 mm and a unit weight of 78.50 kN/m³. Based on relevant specifications, the standard installation torque for field bolts is 350 N·m. Using the torque scaling ratio *C*_*T*_ = 876.32, the corresponding laboratory installation torque was determined to be 0.40 N·m, which produced an axial force of approximately 350 N. Accordingly, a torque wrench was used to apply a consistent pretension at each anchorage position, with the pretension force set to 350 N.

To reduce the influence of specimen-to-specimen variability on the experimental outcomes, all sandstone specimens were cored from the same homogeneous rock block. During specimen preparation, strict consistency was maintained in specimen dimensions, end-face flatness, and the machining procedures for the cavity and prefabricated flaws, thereby minimizing variability attributable to material heterogeneity and manufacturing tolerance. Throughout testing, standardized protocols were applied for bolt installation, loading procedures, and data acquisition to ensure consistent operation and comparability across tests. To rigorously evaluate the effect of anchorage position on the mechanical response of cavity-containing sandstone, two replicate tests were performed for each experimental condition. In addition, two unbolted cavity-containing specimens were prepared as controls to quantify the reinforcement effect of bolting.

### Specimen loading and data acquisition system

**Fig 2:**
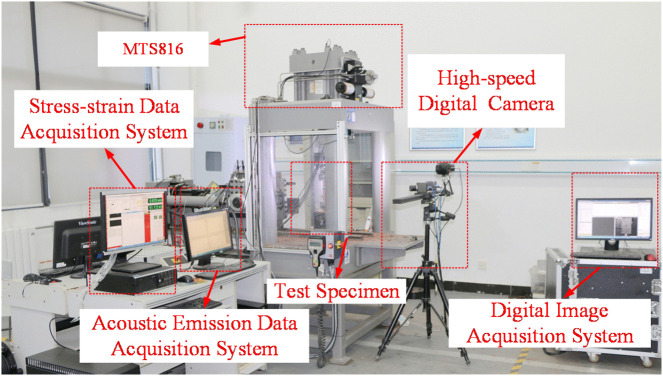
Experimental loading and data acquisition system.

Uniaxial compression tests were performed using an MTS816 rock mechanics testing system (Fig 2). All tests were conducted under displacement control at a loading rate of 0.2 mm/min. The system provides a maximum axial load capacity of 1459 kN and a maximum displacement stroke of 100 mm. To capture surface crack evolution, a high-speed, high-resolution digital image correlation (DIC) system (XTDIC-L) was used concurrently. Images were acquired at an interval of 30 ms per frame, allowing continuous observation from crack initiation and propagation to final coalescence.

A DS2-series acoustic emission (AE) analyzer was used for synchronized monitoring to record parameters including ring-down counts and energy counts. The AE system operated at a sampling rate of 3 MHz with an amplifier threshold of 40 dB, a hit lockout time of 100 µs, a hit definition time of 150 µs, and a peak definition time of 50 µs. Integrating mechanical, visual, and AE datasets enabled a more robust analysis of how bolting suppresses crack propagation.

## Experimental results

### Stress-strain curves

#### Comparison between intact and unbolted cavity specimens.

**Fig 3:**
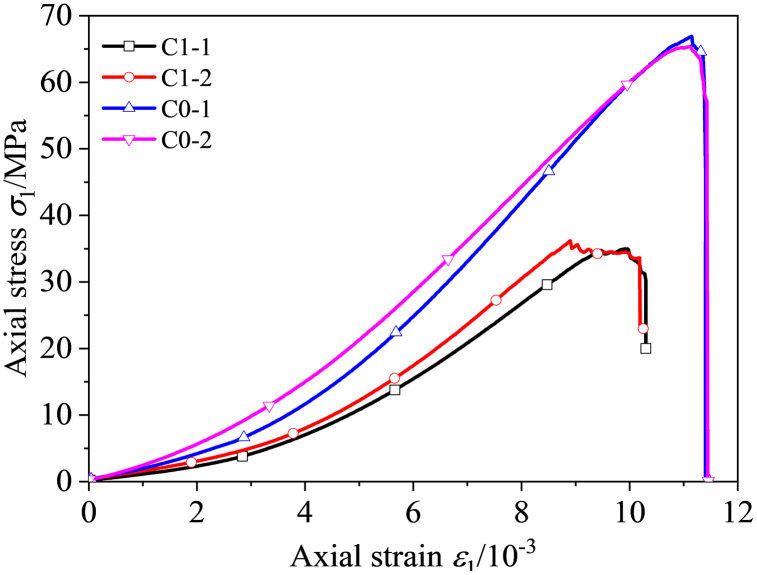
Complete and single-hole specimen stress-strain curves under uniaxial compression.

Fig 3 compares the axial stress-strain responses of intact specimens (C0-1, C0-2) and unbolted specimens with a prefabricated cavity (C1-1, C1-2). Intact specimens showed an average uniaxial compressive strength of 66.14 MPa (coefficient of variation, CV = 2.28%), an average elastic modulus (*E*) of 7.23 GPa (CV = 8.85%), and an average peak strain of 11.14 × 10 ⁻ ³ (CV = 6.10%). In contrast, cavity specimens exhibited marked reductions: compressive strength decreased to 35.58 MPa (CV = 3.34%), *E* to 6.14 GPa (CV = 5.86%), and peak strain to 9.35 × 10 ⁻ ³ (CV = 8.02%).

Within each group, the stress-strain curves were consistent, with all coefficients of variation below 8.85%, indicating good material homogeneity. Introducing a cavity reduced strength by approximately 46%, *E* by 15%, and peak strain by 16%. These results support that the sandstone was relatively uniform and that specimen-to-specimen variability had a limited influence on the measured parameters. This level of consistency supports subsequent comparisons of bolting configurations.

#### Stress-strain curves of bolted specimens.

**Fig 4:**
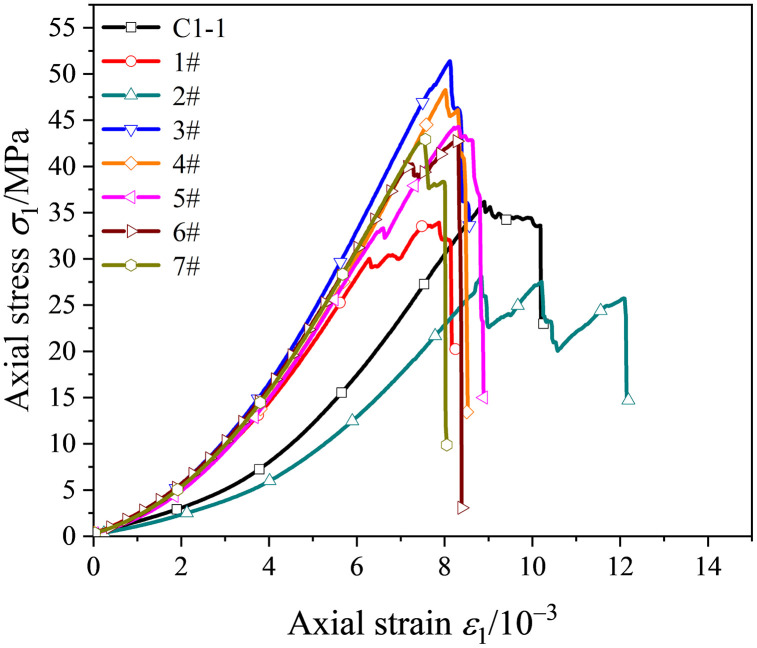
Stress-strain curves of anchored specimens at different locations.

Fig 4 shows representative uniaxial stress-strain curves for specimens bolted at different positions. The curves indicate that anchorage position strongly affects the mechanical response of cavity-containing sandstone. When the bolt intersected the cavity center (position 1#) or was tangent to the cavity crown (position 2#), pronounced stress fluctuations occurred both before and after peak stress. This behavior is attributed to overlap between the bolt borehole and the prefabricated cavity, which creates a localized damage concentration zone. Cracks preferentially propagated within this zone during loading, resulting in reduced strength and pronounced mechanical degradation. Degradation was most pronounced at position 2#.

As the bolt-hole distance (*l*) increased, strength and stiffness first increased and then decreased. A pronounced strengthening effect was observed at *l* = 12 mm (3#) and *l* = 18 mm (4#), with substantial gains in strength and stiffness. In addition, post-peak brittleness was reduced and deformation capacity increased. Among all configurations, position 3# (*l* = 12 mm) provided the strongest overall improvement in compressive performance (and associated shear resistance). With further increases in *l*, load-bearing capacity gradually decreased and then approached a stable level.

**Fig 5:**
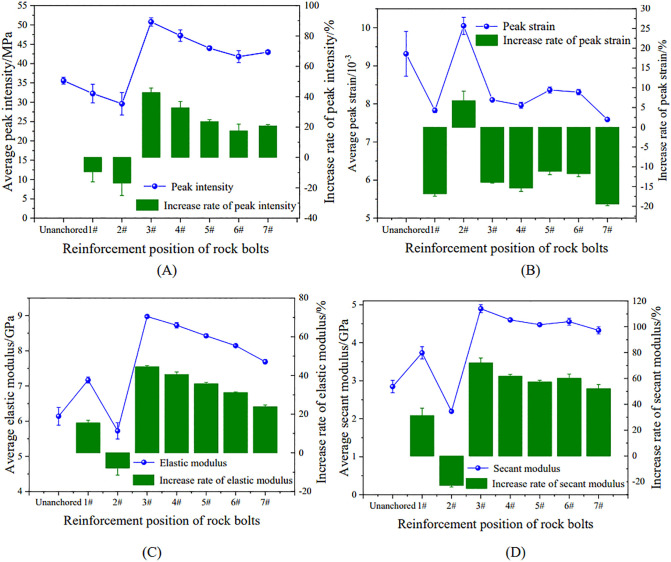
Characteristic laws of mechanical parameters for specimens. (A) peak strength; (B) peak strain;(C) *E*; (D) secant modulus.

Systematic uniaxial compression tests were conducted to quantify key mechanical parameters, including peak strength and *E*, for specimens with different bolt positions (Table 1). Fig 5 further illustrates how bolt position governs the strength and deformation responses of the specimens.

**Table 1:** Uniaxial compressive mechanical parameters of specimens.

Bolt position	Specimen number	Peak strength/MPa	Mean peak strength/MPa	Peak strain/10^–3^	Mean peak strain/10^–3^	Elastic modulus/MPa	Mean elastic modulus/MPa	Secant modulus/MPa	Mean secant modulus/MPa
**Unanchored**	C1-1	34.97	35.58	9.73	9.42	5.96	6.21	2.73	2.85
	C1-2	36.19		8.98		6.32		2.96	
**1#**	C2-1–1	33.94	32.25	7.87	7.83	7.11	7.17	3.85	3.74
	C2-1–2	30.55		7.79		7.23		3.62	
**2#**	C3-1–1	27.53	29.61	10.21	10.05	5.56	5.725	2.22	2.20
	C3-1–2	31.68		9.89		5.89		2.17	
**3#**	C4-1–1	51.54	50.78	8.12	8.11	9.00	8.98	4.97	4.90
	C4-1–2	50.02		8.09		8.95		4.82	
**4#**	C5-1–1	48.27	47.23	8.02	7.97	8.78	8.73	4.57	4.60
	C5-1–2	46.19		7.91		8.67		4.63	
**5#**	C6-1–1	44.31	43.98	8.31	8.37	8.46	8.43	4.50	4.48
	C6-1–2	43.65		8.42		8.39		4.45	
**6#**	C7-1–1	42.92	41.81	8.26	8.31	8.13	8.15	4.49	4.56
	C7-1–2	40.69		8.36		8.16		4.62	
**7#**	C8-1–1	42.74	42.98	7.56	7.59	7.73	7.69	4.39	4.33
	C8-1–2	43.21		7.62		7.65		4.26	

As shown in Fig 5A, anchorage position strongly regulated peak strength. When the bolt was placed at the cavity center (1#) or tangent to the cavity crown (2#), no strengthening was observed; instead, peak strength decreased by 9.37% and 16.79%, respectively. In contrast, for positions 3#–7#, peak strength increased initially with distance, then declined, and finally stabilized. Position 3# (*l* = 12 mm) produced the largest strengthening effect, with an average peak strength of 50.78 MPa (42.72% higher than the unanchored specimen) and recovery to 76.78% of the intact-specimen strength. This suggests that anchoring at 3# most effectively suppressed crack propagation around the cavity, thereby maximizing strength improvement.

Fig 5B summarizes the effects of anchorage position on deformation behavior. Position 2# exhibited the largest peak strain (10.05 × 10 ⁻ ³), 6.74% higher than the unanchored specimen (9.42 × 10 ⁻ ³), indicating greater deformability. However, strength at this position decreased, implying that the larger deformation reflected plastic yielding driven by localized damage concentration rather than improved load capacity. Peak strains at all other anchorage positions were lower than the unanchored specimen, with reductions of 11.15%–19.43%. Together with the strength and stiffness gains, these results indicate that an optimized anchorage layout can limit excessive deformation by suppressing crack growth, thereby improving overall mechanical performance.

Bolt position markedly affects the elastic and secant moduli by altering crack propagation behavior. As shown in Fig 5C and 5D, both moduli vary nonlinearly with increasing bolt distance (*l*), decreasing initially, then increasing, and finally decreasing again, mirroring the trend in peak strength.

Specimens anchored at position 2# showed the lowest stiffness, with elastic and secant moduli reduced by 7.81% and 22.85%, respectively, relative to the unanchored specimens. In contrast, position 3# produced the greatest stiffness enhancement, increasing the elastic and secant moduli by 44.61% and 71.93%, respectively. Notably, the *E* at position 3# reached 1.24 times that of the intact specimens, indicating that this configuration best preserves structural stiffness. These coordinated trends provide quantitative guidance for optimizing bolt placement to mitigate crack propagation.

### Analysis of fracture evolution characteristics

Acoustic emission (AE) monitoring is widely used to track rock fracturing and characterize crack evolution in defective rock masses. Prior studies show that AE signals can capture key features of internal fracture mechanisms. For example, Zhou et al. [47] analyzed AE parameters to distinguish tensile and shear failure mechanisms in sandstone with double fissures under uniaxial compression. Liu et al. [48] reported that, for sandstone with an elliptical cavity, stress fluctuations corresponded closely to inflection points in the cumulative AE count curve. However, AE characteristics during loading remain insufficiently documented for composite systems such as bolted sandstone with a cavity. Accordingly, we used real-time AE monitoring to characterize fracture evolution in bolted sandstone containing a single hole, with emphasis on how bolt position suppresses crack propagation.

**Fig 6:**
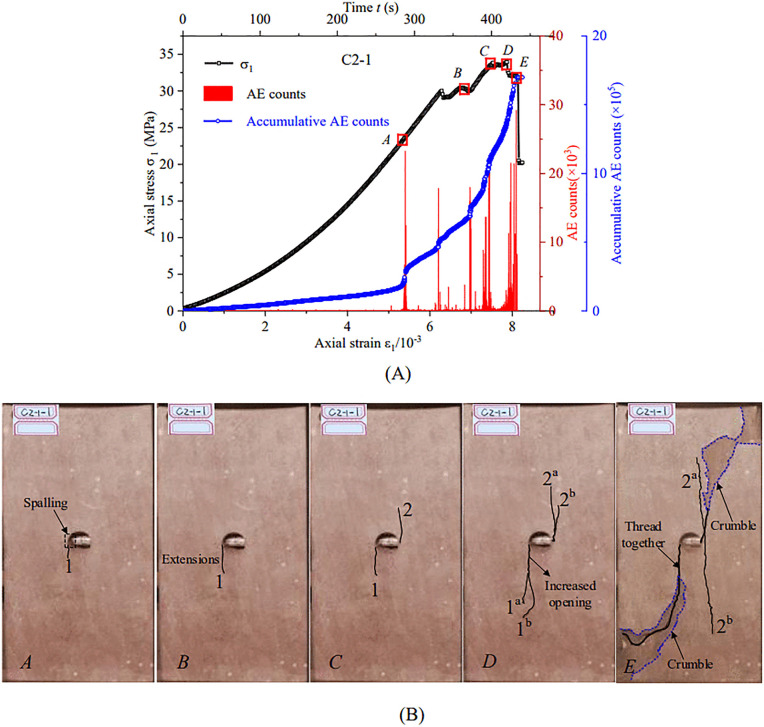
AE distribution curves and fracture evolution process of anchored specimen at Position 1#. **(A)** Stress-strain behavior and AE characteristics of anchored specimen 1#; **(B)** Crack propagation and failure mechanism of the anchored sample from position 1#.

To illustrate the effect of bolt position on crack evolution, a representative specimen anchored at position 1# was selected for detailed analysis. We examined its AE activity (Fig 6A), axial stress-strain response, and crack evolution sequence (Fig 6B). During early loading (Point *A*; *σ*_1_ = 23.15 MPa, *ε*_1_ = 5.31 × 10 ⁻ ³), a tensile crack (Crack 1) initiated at the intersection between the left side of the cavity and the bolt borehole, coinciding with pronounced AE activity. At Point *B* (*σ*_1_ = 30.35 MPa), Crack 1 propagated and AE signals intensified. At Point *C* (*σ*_1_ = 33.77 MPa), a reverse tensile crack (Crack 2) initiated on the right side of the cavity. After the peak at Point *D* (*σ*_1_ = 33.94 MPa), Cracks 1 and 2 rapidly extended and produced secondary cracks (1^a^, 1^b^, 2^a^, and 2^b^). AE events became dense and the cumulative AE curve rose sharply, indicating entry into an accelerated damage stage. As stress decreased to Point *E* (*σ*_1_ = 32.06 MPa), the primary cracks (Cracks 1 and 2) coalesced with the free surface. Elastic energy release triggered spalling accompanied by high-intensity AE bursts. Ultimately, the specimen became unstable and stress dropped to 20.49 MPa. The cumulative AE curve increased almost vertically, consistent with complete loss of load-bearing capacity.

### Crack initiation modes and initiation stress

**Fig 7:**
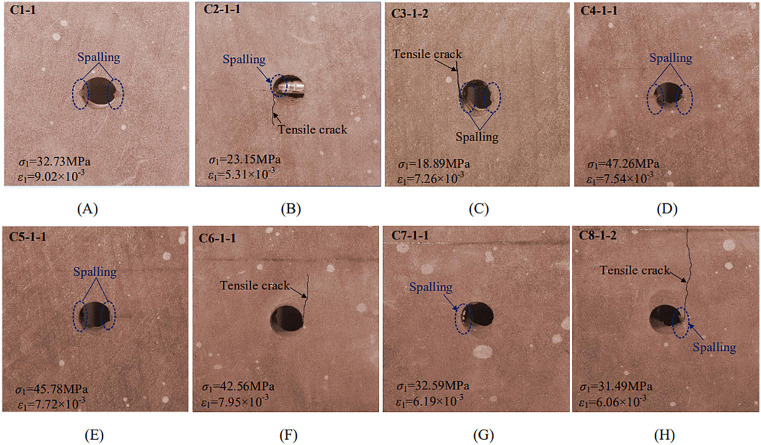
Initial crack initiation stress-strain conditions and methods in specimens. (A) unanchored; (B) position 1#; (C) position 2#; (D) position 3#; (E) position 4#; (F) position 5#; (G) position 6#; (H) position 7#.

Fig 7 summarizes crack initiation modes for each specimen together with the corresponding stress-strain states. These observations allow systematic assessment of how bolt position governs crack initiation. In the unanchored specimen, initial damage occurred at a vertical stress of 32.73 MPa as spalling developed on both lateral sides of the cavity (Fig 7A), indicating that stress concentration was localized near the cavity boundary.

In bolted specimens, crack initiation showed clear spatial dependence on bolt location. When the bolt borehole intersected the cavity (1#) or was tangent to it (2#), specimens exhibited wall spalling together with a tensile crack initiating approximately tangent to the cavity wall. This tensile crack then propagated along the loading direction toward the specimen end (Figs 7B and 7C). In this overlap or near-overlap zone, interference between the bolt borehole and the cavity produced localized weakening, and initiation was dominated by crack nucleation and subsequent propagation. For positions 3# and 4#, initiation primarily manifested as wall spalling on both sides of the cavity (Figs 7D and 7E). At position 5#, wall spalling was accompanied by a microcrack (Fig 7F), whereas positions 6# and 7# still exhibited wall spalling as the dominant initiation feature (Fig 7G and 7H).

Overall, when the bolt borehole intersected or was tangent to the cavity, the anchorage zone experienced strength weakening, and initiation was governed mainly by tensile crack nucleation and growth. As the bolt-cavity distance increased, stress superposition diminished and initiation modes became more uniform, with spalling dominating.

**Fig 8:**
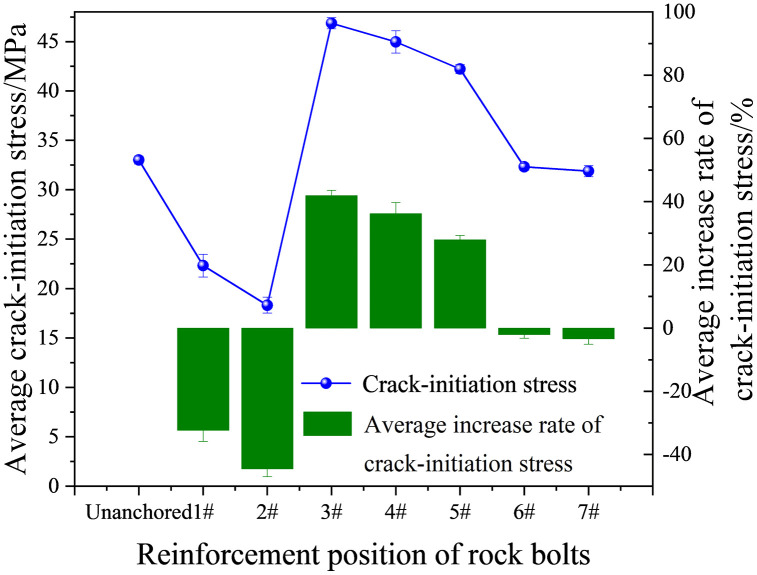
Trends in specimen crack initiation stress.

Using high-resolution images and stress measurements, Fig 8 shows the relationship between crack initiation stress and bolt position. To quantify the anchorage effect, we computed the relative change in initiation stress for each position using the unanchored specimen as the reference. Crack initiation stress and its relative change followed trends consistent with other mechanical indicators, including peak strength and *E*.

Positions 1# and 2# substantially reduced crack initiation stress. At position 2#, the mean initiation stress decreased to 17.74 MPa, a 46.73% reduction relative to the unanchored specimen. In contrast, position 3# produced the strongest improvement, with a mean initiation stress of 47.26 MPa (39.53% higher than the unanchored specimen). With further increases in bolt-cavity distance, initiation stress decreased overall, with particularly pronounced reductions at positions 6# and 7#. This pattern may be explained by changes in the stress-transfer path. When the bolt is placed closer to the loading end, the effective stress-transfer path becomes shorter. Together with localized damage concentration introduced by the bolt borehole, these effects promoted earlier crack initiation and premature failure.

### Failure mode analysis

**Fig 9:**
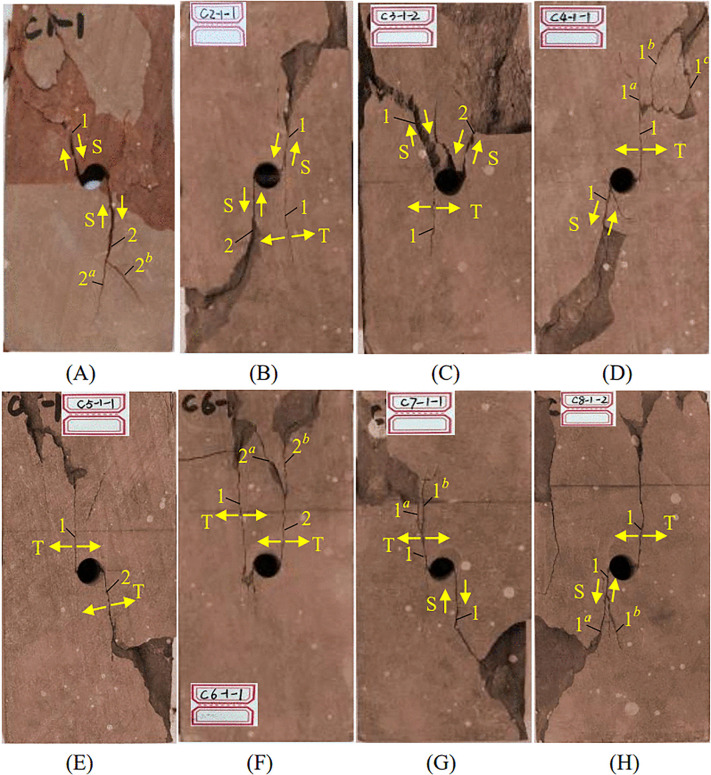
Fracture mode of the sample. (A) unanchored; (B) position 1#; (C) position 2#; (D) position 3#; (E) position 4#; (F) position 5#; (G) position 6#; (H) position 7#.

**Fig 10:**
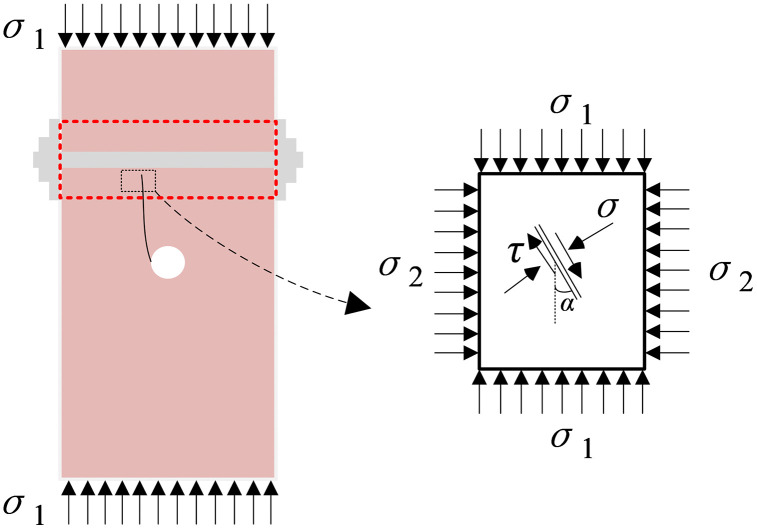
Mechanical model of sliding cracks within the bolted zone.


Bolt position modifies the internal stress distribution and therefore governs crack propagation in cavity-containing sandstone [49,50]. Fig 10 shows the post-failure morphologies of specimens for each bolting configuration. The unanchored cavity specimen exhibited a comparatively simple failure pattern, dominated by two main shear cracks (Cracks 1 and 2) initiating on opposite sides of the cavity and propagating toward the loading ends; Crack 2 further produced secondary shear branches (2^a^ and 2^b^) (Fig 9A). Once the main shear crack connected to the free surface, rapid energy release induced extensive spalling and led to overall shear instability.

Specimens bolted at positions 1# and 2# remained shear-dominated but showed distinct features. At position 1#, the two main shear cracks remained on opposite sides of the cavity, a companion tensile crack developed during propagation, and spalling was substantially reduced; the bolt acted as a dowel that suppressed abrupt energy release, with localized block breakage occurring mainly at the final instability stage (Fig 9B). At position 2#, both main shear cracks were concentrated on the same side of the cavity; pronounced crown collapse occurred and the fracture surface formed an approximately V-shaped pattern (Fig 9C). This behavior is attributed to superposition of stress fields from the bolt borehole and cavity, which intensified stress concentration at the crown. The lower region remained relatively intact with only fine tensile cracks, suggesting an altered stress-transfer path.

The 3# specimen showed clear spatially differentiated failure separated by the cavity, with a transition to tensile-dominated cracking in the upper region and shear-dominated cracking in the lower region (Fig 9D). For positions 4# and 5#, tensile cracking dominated both above and below the cavity, accompanied by localized microcracking and flake-like spalling, and overall specimen integrity was better preserved (Figs 9E and 9F).

Similar to 3#, specimens at 6# and 7# exhibited spatial differentiation, but their failure morphologies were more complex, with more fragmented blocks and greater overall damage (Figs 9G and 9H). This suggests that beyond a critical bolt distance, the crack-restraint effect weakens and failure becomes a more dispersed tensile-shear composite mode. Collectively, these failure transitions demonstrate that bolt position controls failure mechanisms and stability in cavity-bearing rock, providing a basis for evaluating support effectiveness in suppressing crack growth.

## Analysis of bolt anchoring mechanism for crack arrest

Under uniaxial loading, the bolt is primarily subjected to tensile forces, shear forces, and their coupled actions. Accordingly, the crack-arresting role of bolting can be interpreted via two mechanisms: an axial-compression effect induced by bolt tension and a tangential anchoring effect associated with bolt shear. In this study, the bolt-hole diameter was designed to match the bolt diameter. After installation, only a negligible clearance remained between the bolt and the borehole wall. Before global instability occurs, the contact force generated by this small clearance between the bolt and the borehole wall is minimal and can be neglected. Therefore, to simplify the formulation and isolate the axial-compression mechanism, shear effects, bonding/adhesion, and complex bolt-rock contact interactions are not explicitly considered in the present analysis.

As shown in Fig 10, following the classical sliding-crack model [51–53], frictional resistance (*τ*_*f*_) is assumed to act along the fracture surfaces of compression-shear cracks in the bolted sandstone. The frictional behavior is described using the Mohr-Coulomb criterion. The effective shear stress on the fracture surfaces, *τ*_*fi*_ [54], is expressed as:

$$\tau_{fi}=\tau_{xy}-\tau_{f}=\tau_{xy}-\mu_{f}\sigma_{n}$$ (5)

where *τ*_*xy*_ denotes the shear stress on the fracture surface, *σ*_*n*_ is the normal stress, and *μ*_*f*_ is the Coulomb friction coefficient.

The normal stress *σ*_*n*_ and shear stress *τ*_*xy*_ can be written as:


$$\left\{ \begin{array}{c} \sigma_{n}=\frac{\text{1}}{\text{2}}\left[(\sigma_{\text{1}}+\sigma_{\text{2}})-(\sigma_{\text{1}}-\sigma_{\text{2}})\text{cos}(\text{2}\alpha )\right]\\ \tau_{xy}=\frac{\text{1}}{\text{2}}(\sigma_{\text{1}}-\sigma_{\text{2}})\text{sin}(\text{2}\alpha ) \end{array}\right.\;$$ (6)

Substituting Eq 6 into Eq 5 gives:


$$\tau_{fi}=\frac{\text{1}}{\text{2}}(\sigma_{\text{1}}-\sigma_{\text{2}})\left[\text{sin}(\text{2}\alpha )+\mu_{f}\text{cos}(\text{2}\alpha )\right]-\frac{\text{1}}{\text{2}}\mu_{f}(\sigma_{\text{1}}+\sigma_{\text{2}})$$ (7)

The sliding-crack model posits that when the far-field loading induces shear stress exceeding the maximum shear resistance (*τ*_*c*_) on the fracture surface (i.e., *τ*_*fi*_ > *τ*_*c*_), relative sliding occurs, thereby triggering the initiation and propagation of wing cracks at the crack tips.

The stress-strain responses and observed failure patterns indicate that, with progressive axial shortening and lateral dilation, the bolt axial force increases gradually, which in turn elevates the lateral (horizontal) stress *σ*_2_ within the bolted rock. According to Eq 7, an increase in *σ*_2_ markedly decreases the effective shear stress *τ*_*fi*_ on the fracture surface, thereby suppressing further crack propagation and improving the overall load-bearing capacity of the specimen. Moreover, increasing the bolt pretension further enhances specimen strength and correspondingly mitigates internal damage.

**Fig 11:**
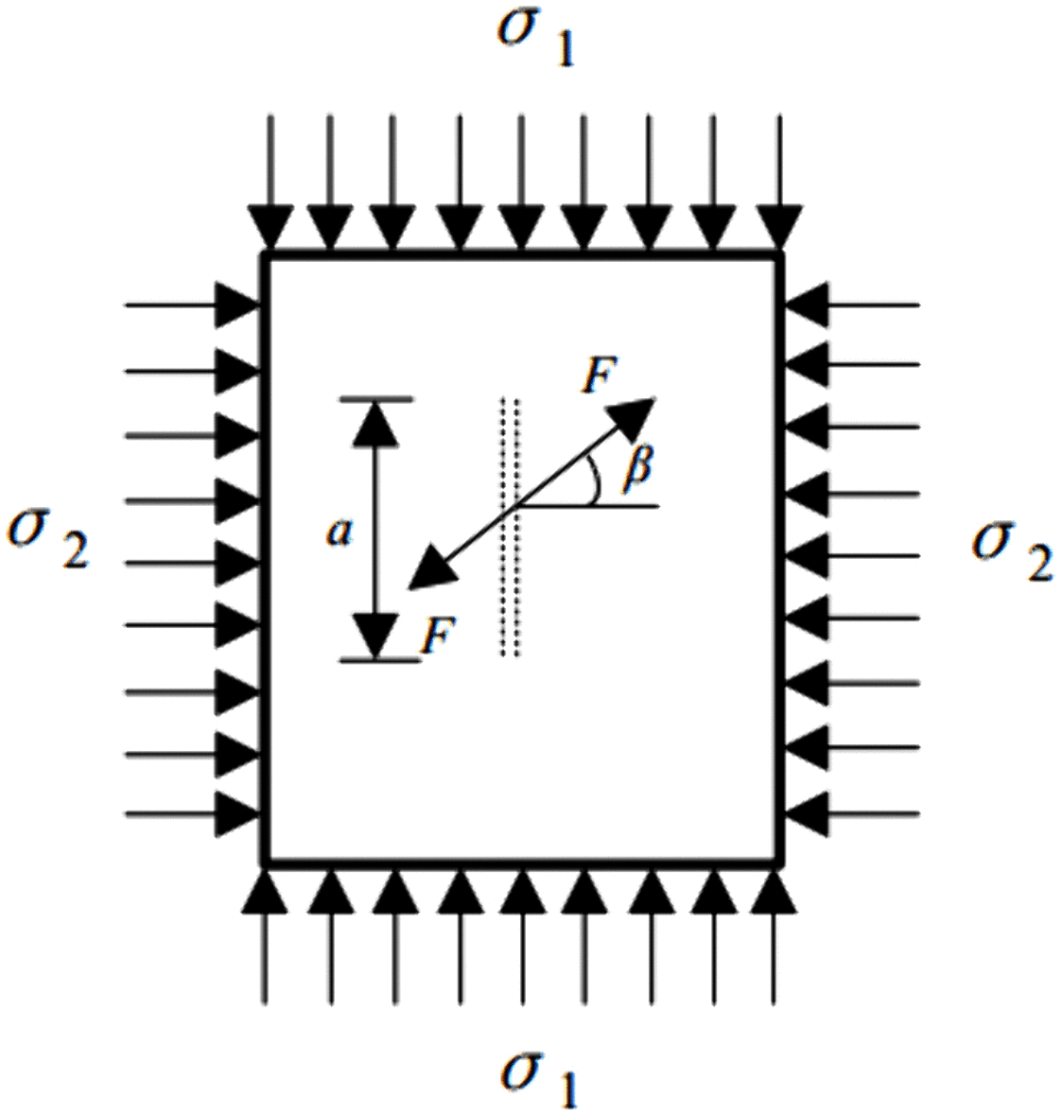
Equivalent crack model of wing cracks in the anchored region.


Within the anchored zone, wing cracks are predominantly tensile and can be idealized as an equivalent crack system (Fig 11). Specifically, the two wing cracks are equivalently represented as a single collinear crack aligned with the maximum compressive stress direction (*σ*_1_). The wing crack has a propagation length of *a*, while the main crack has *a* length of *b*. The effect of the main crack is accounted for by a pair of collinear concentrated forces *F* = 2*bτ*_*fi*_ applied at the center of the equivalent crack. Under the combined action of the far-field stresses *σ*_1_ and *σ*_2_, along with the shear driving force *F*, the stress intensity factor *K* at the the wing-crack tip is given by [55]:

$$K=\frac{F\text{cos}\beta }{\sqrt{\pi a}}-\sigma_{\text{2}}\sqrt{\pi a}$$ (8)

where *β* is the angle between the concentrated force *F* and the horizontal stress *σ*_2_.

As suggested by Eq 8, bolt prestressing (i.e., an increase in *σ*_2_) reduces the crack-tip stress intensity factor, thereby retarding wing-crack propagation. Consequently, further wing-crack extension requires a higher applied axial stress *σ*_1_. In addition, continued wing-crack growth produces progressive opening displacement between the fracture surfaces. This opening promotes a faster increase in bolt axial force, which further strengthens the crack-arresting capacity of the anchoring system.

## PFC^2D^ numerical analysis

### Numerical model and determination of meso-scale parameters

**Fig 12:**
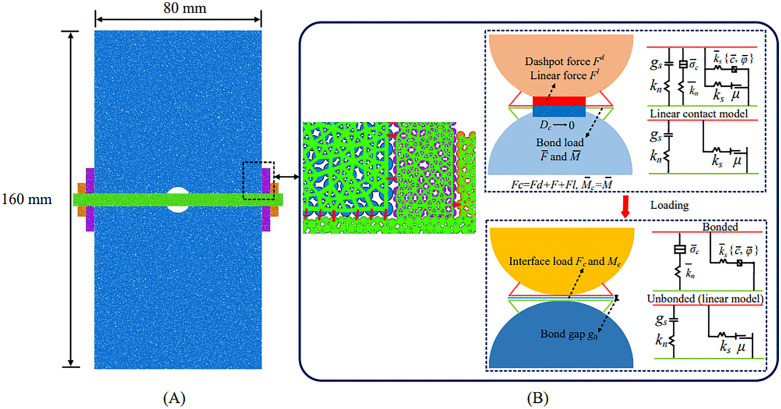
PFC^2D^ numerical calculation model. (A) numerical model; (B) contact model.

Particle Flow Code 2D (PFC^2D^) is a discrete-element method (DEM) tool widely used to simulate the mechanical behavior of rock. PFC^2D^ can capture the linkage between meso-scale structural evolution and macro-scale mechanical response, and is therefore suitable for simulating diverse experimental conditions [56,57]. The numerical specimen was modeled with dimensions of 160 mm × 80 mm. Red sandstone was used as the modeled material. The minimum and maximum particle radii were set to 0.10 mm (*R*_min_) and 0.14 mm (*R*_max_), respectively, yielding *R*_max_/ *R*_min_ = 1.40. Particle radii were assigned using a uniform distribution The model comprised 25,907 randomly generated particles, resulting in 67,771 contacts. The model density was set to 2560 kg/m³ (Fig 12A).

Among the built-in contact models in PFC^2D^, the parallel bond model can reproduce key aspects of rock failure mechanisms. Because no adhesive was used in the experiments and the bolt fit tightly within the borehole, the bolt–rock interface was simplified as a frictional physical contact. Normal stiffness, shear stiffness, and the friction coefficient were prescribed directly at the bolt–rock interface. Accordingly, the parallel bond model was used to represent both the sandstone matrix and the bolt–borehole contact interface (Fig 12B). This setup was adopted to better capture the failure processes and mechanical responses under different anchoring positions.

**Fig 13:**
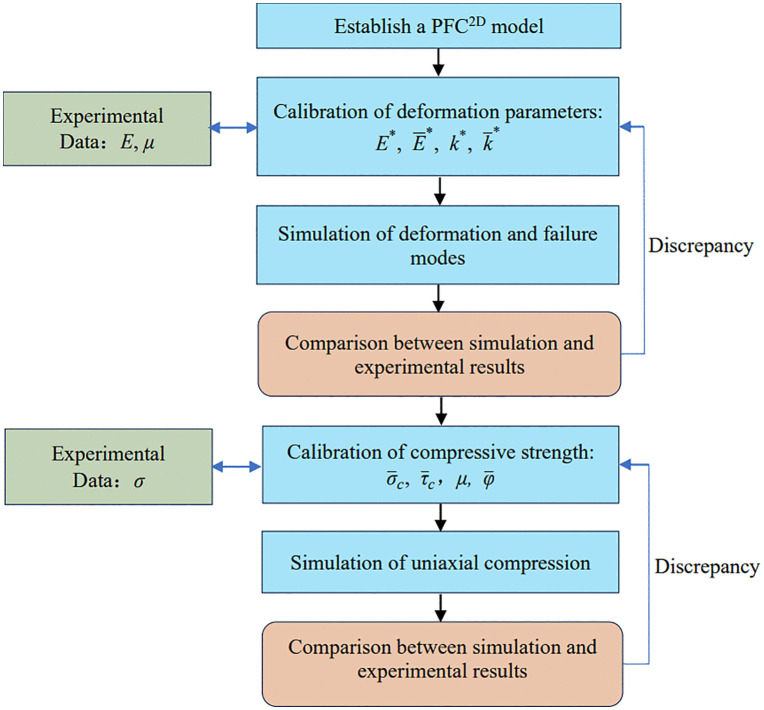
Flowchart of meso-scale parameter calibration.

Using results from laboratory uniaxial compression tests and bolt pull-out tests, meso-scale parameters were calibrated iteratively via a trial-and-error procedure. Calibration was performed sequentially, first for intact sandstone and then for the bolt. A holed, anchored specimen model was then constructed, and the calibrated parameters were applied to the sandstone and bolt systems, respectively. The calibration workflow is summarized in Fig 13. Calibrated meso-scale parameters are reported for sandstone in Table 2 and for the bolt, bearing plate, nut, and anchorage interface in Table 3.

**Table 2:** Microscopic parameters for the PFC^2D^ sandstone specimen simulation.

Model parameter	Notation	Value	Model parameter	Notation	Value
**Minimum particle radius/mm**	*r*	0.10	**Parallel bond stiffness ratio**	${\overset{―}{k}}^{*}$	2.50
**Particle size ratio**	*λ*	1.40	**Normal bond strength/MPa**	${\overset{―}{\sigma }}_{c}$	120.60
**Particle density/ (kg/m³)**	$\overset{―}{\rho }$	2550	**Tangential bond strength/MPa**	${\overset{―}{\tau }}_{c}$	170.50
**Particle contact modulus/GPa**	$E^{*}$	3.05	**Friction angle/°**	$\overset{―}{\varphi }$	30
**Particle stiffness ratio**	$k^{*}$	1.70	**Friction coefficient**	*μ*	0.50
**Parallel bond effective modulus/GPa**	${\overset{―}{E}}^{*}$	3.05	_	_	_

**Table 3:** Mesoscopic parameters for the simulation of bolting materials in PFC^2D^.

Parameters for bolt, plate & nut	Notation	Value	Parameters for bolted interface	Notation	Value
**Maximum particle radius/mm**	*r*	0.20	**Linear deformation modulus/GPa**	*E*	1.00
**Particle contact modulus/GPa**	$E^{*}$	10.25	**Parallel bond modulus/GPa**	${\overset{―}{E}}^{*}$	165
**Particle stiffness ratio**	$k^{*}$	2.30	**Parallel bond stiffness ratio**	${\overset{―}{k}}^{*}$	2.00
**Linear deformation modulus/GPa**	*E*	1.20	**Parallel bond tensile strength/MP**	${\overset{―}{\sigma }}_{t}$	900.00
**Parallel bond modulus/GPa**	${\overset{―}{E}}^{*}$	195	**Parallel bond shear strength/MPa**	${\overset{―}{\tau }}_{c}$	900.00
**Parallel bond stiffness ratio**	${\overset{―}{k}}^{*}$	1.80	**Parallel bond internal friction angle**	$\overset{―}{\varphi }$	46°
**Particle friction coefficient**	*μ*	0.95	**Friction coefficient**	*μ*	0.90

**Fig 14:**
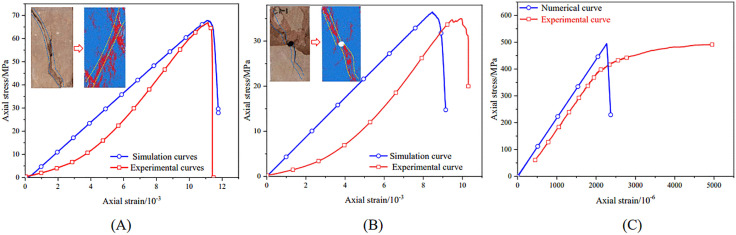
Comparison between physical experiments and numerical simulation results. (A) uniaxial compression test of intact specimen;(B) uniaxial compression test of specimen with a hole defect;(C) bolt pull-out test.

Fig 14 compares laboratory and numerical stress–strain responses from uniaxial compression tests (intact and holed specimens) and bolt pull-out tests. For the intact specimen (Fig 14A), the laboratory and simulated peak strengths were 66.89 MPa and 67.82 MPa, respectively (difference: 1.37%). The corresponding elastic moduli were 7.23 GPa and 6.89 GPa (difference: 4.70%). For the holed specimen (Fig 14B), the laboratory-measured and simulated peak strengths were 34.97 MPa and 35.43 MPa (difference: 1.32%), and the elastic moduli were 6.21 GPa and 6.05 GPa (difference: 2.59%). In both cases, the dominant failure mode was shear failure. For the bolt pull-out test (Fig 14C), the *E* in the linear-elastic stage was 196.65 GPa in the laboratory measurements and 199.37 GPa in the simulations, corresponding to a difference of 1.38%.

Overall, the PFC^2D^ simulations reproduced the experimentally measured macro-scale parameters and dominant failure modes, supporting the suitability of the calibrated meso-scale parameters.

### Simulation results of mechanical characteristics

After validating the model, we systematically evaluated how anchoring position influences mechanical response, crack evolution, and bolt axial force in holed sandstone, with emphasis on crack-propagation inhibition. Numerical anchoring configurations matched the laboratory schemes to enable direct comparisons.

**Fig 15:**
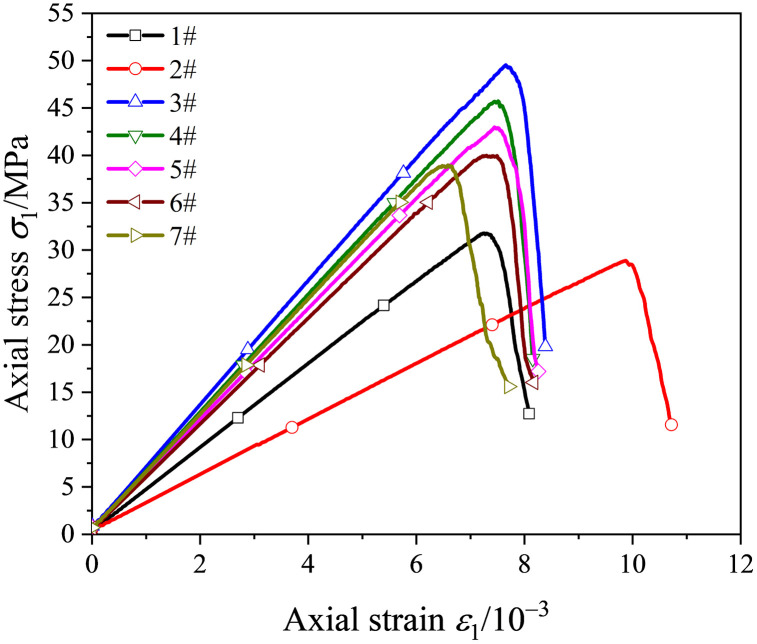
Stress-strain curves of numerical specimens containing a hole defect anchored at different positions.

Fig 15 shows the simulated stress–strain curves for seven anchoring positions. As in the intact and holed numerical specimens, the simulated curves did not exhibit a distinct initial compaction stage, in contrast to the laboratory results. In the post-peak regime, all anchored specimens showed pronounced plastic deformation. This response suggests that anchorage restrained crack propagation and coalescence, thereby shifting the failure process from abrupt brittle failure toward a more progressive, ductile-like response.

**Fig 16:**
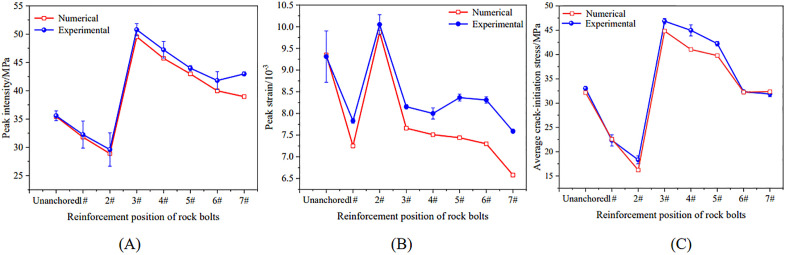
Variation patterns of peak strength, peak strain, and crack initiation stress in numerical specimens with a hole defect anchored at different positions. (A) peak strength; (B) peak strain; (C) crack initiation stress.

To quantify anchoring-position effects, Fig 16 summarizes peak strength, peak strain, and crack initiation stress across numerical specimens.

Peak strength varied non-monotonically with anchoring position, showing a decrease–increase–decrease pattern. As shown in Fig 16A, when the bolt passed through the hole center (position 1#, 31.78 MPa) or was tangent to the upper edge of the hole (position 2#, 28.89 MPa), the peak strength was relatively low, indicating a limited strengthening effect. Relative to the mean peak strengths from the laboratory tests, the corresponding errors were 1.46% and 2.43%, respectively. When the anchorage position was adjusted to position 3#, the peak strength increased to a maximum of 49.53 MPa (error: 2.54%). With further movement of the anchorage position away from the hole, the peak strength decreased approximately linearly, reaching 38.95 MPa at the farthest position, with an error of 4.28%. This trend was consistent with the laboratory results, with both datasets showing the maximum strength at position 3#. The small discrepancies between simulations and experiments (1.46%–4.28%) further support model reliability.

In contrast to peak strength, peak strain exhibited an “increase-then-decrease” trend with anchorage position (Fig 16B). The specimen at position 2# showed the largest peak strain (9.87 × 10 ⁻ ³), indicating greater inelastic deformation capacity, whereas the specimen at position 7# (farthest from the hole) exhibited the smallest peak strain (6.58 × 10 ⁻ ³), reflecting the weakest ductile-like response among the anchored cases. Simulated peak strains were generally lower than those measured experimentally. This difference likely reflects the idealized nature of the PFC^2D^ particle assembly, which assumes initially well-bonded particles and does not explicitly capture the initial compaction stage or randomly distributed pre-existing microdefects. As a result, the early-stage response is more linearized, leading to smaller simulated peak strains.

Crack initiation stress also followed a decrease–increase–decrease trend with anchorage position (Fig 16C), broadly consistent with the laboratory observations. Across anchorage positions, crack initiation stress ranged from 16.21 MPa (position 2#) to 44.82 MPa (position 3#), with errors relative to experimental values of 0.25%–9.58%. Although crack initiation stress did not vary monotonically, position 3# is notable because it simultaneously produced the highest peak strength and the highest crack initiation stress. This indicates that anchoring at position 3# not only increased load-bearing capacity but also substantially enhanced resistance to crack initiation, yielding the most favorable overall reinforcement effect.

**Fig 17:**
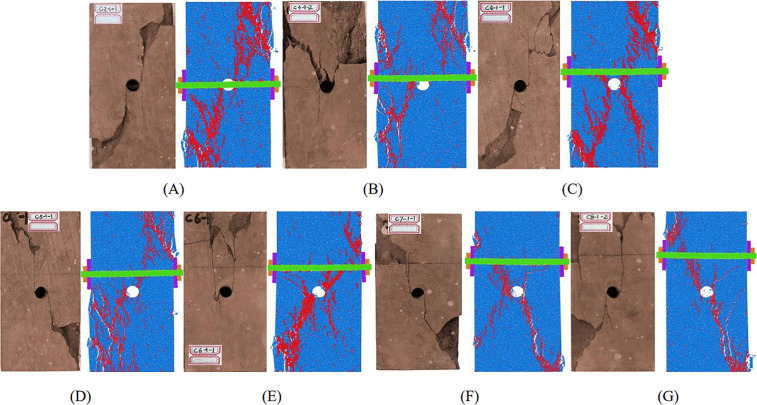
Comparison of failure modes between specimens from laboratory tests and numerical simulation. (A) position 1#;(B) position 2#;(C) position 3#;(D) position 4#;(E) position 5#;(F) position 6#;(G) position 7#.

Fig 17 shows that the simulated failure morphologies for the seven anchorage positions were in good agreement with the laboratory observations. Overall, the dominant failure mode transitioned from primarily shear failure at positions 1# and 2# (Figs 17A and 17B) to a tensile–shear mixed mode at positions 3#–7# (Figs 17C–G). Notably, specimens anchored at position 3# exhibited the most effective crack suppression and the latest onset of macroscopic failure in both the experiments and simulations, consistent with the superior mechanical performance described above. Although minor differences were observed in the development of local secondary cracks, the model reproduced the key features of anchorage-controlled fracture evolution, further supporting its suitability for anchorage-position optimization.

Based on quantitative and qualitative comparisons between the numerical results and the mechanical responses measured in the laboratory, the model developed in this study showed good agreement with the physical experiments. This agreement supports the validity of the numerical model and its predictive capability for investigating the underlying mechanisms.

### Analysis of crack and bolt axial force evolution

To elucidate how anchoring position suppresses crack propagation, we used PFC^2D^ to track meso-scale damage (microcrack initiation, growth, and coalescence) and related it to the evolution of bolt axial force.

**Fig 18:**
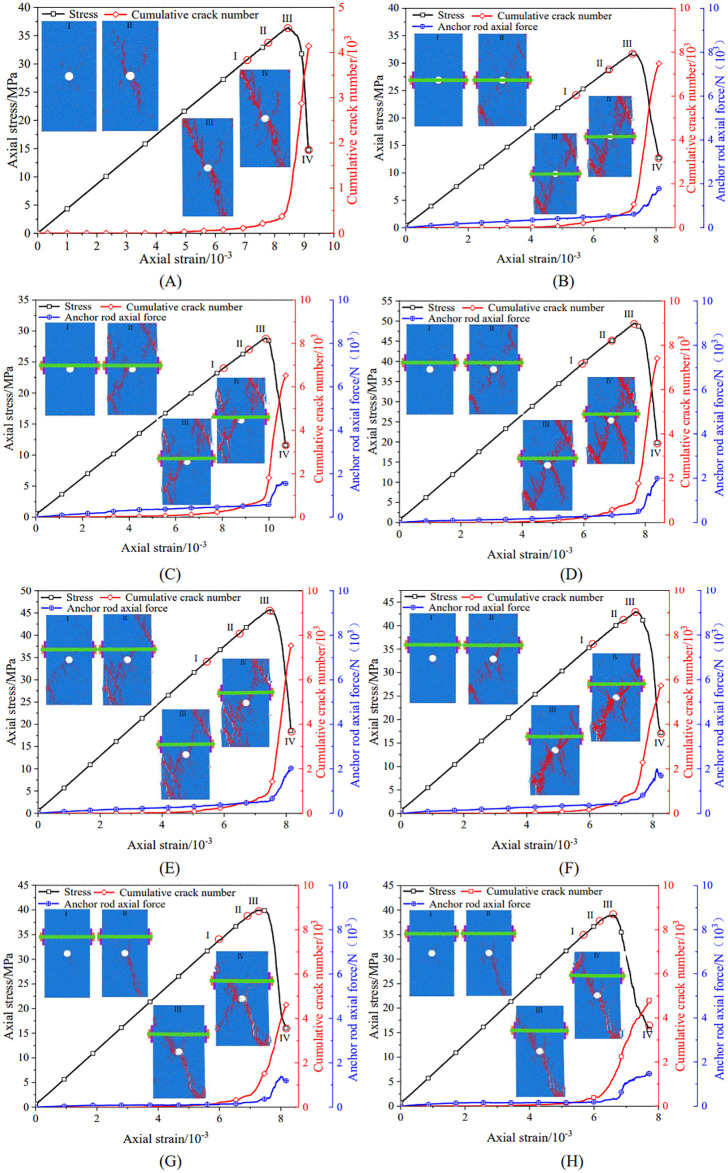
Evolution trends of the total number of cracks and bolt axial force in the specimens. (A) unanchored specimen with a hole defect;(B) position 1#;(C) position 2#;(D) position 3#;(E) position 4#;(F) position 5#;(G) position 6#;(H) position 7#.

Figs 18A–H summarize the relationships among stress–strain response, cumulative microcrack count, and bolt axial force for eight numerical cases. Four stages (I–IV) were identified: elastic deformation, crack propagation, peak load, and post-peak residual response. Macroscopic morphologies are shown for each stage (blue: sandstone particles; red: microcracks). Together, these outputs visualize the meso- to macro-scale correspondence between crack evolution and mechanical response.

To clarify the anchoring mechanism, positions 2# and 3# were selected as representative cases for comparison. For the 2# specimen (Fig 18C), during the pre-peak elastic stage (Point I, *σ* = 23.71 MPa, *ε* = 7.95 × 10 ⁻ ³), only 261 microcracks formed, which were predominantly tensile (256 cracks) and concentrated in the upper-left region at the hole–bolt interface. With increasing load, cracks preferentially propagated toward regions outside the anchored zone, particularly the lower-left side of the hole and the free area above the bolt (from Point II to Point III). By the residual stage (Point IV, *σ* = 11.57 MPa), the crack count increased sharply to 6,859, and extensively coalesced damage bands developed. Because cracking initiated early and propagated outside the anchored zone, deformation restraint was limited, leading to a gradual axial-force increase and a moderate peak force.

By contrast, position 3# exhibited stronger crack-control behavior (Fig 18D). During the pre-peak stage (Point I, *σ* = 24.28 MPa, *ε* = 5.15 × 10 ⁻ ³), microcracks were confined to the vicinity of the bolt borehole (161 cracks). Even in the residual stage (Point IV, *σ* = 19.83 MPa), although the total crack count reached 7,787, the formation of new cracks within the anchored zone remained markedly constrained. This crack-diversion and shielding effect transferred a larger fraction of deformation-induced load to the bolt. Accordingly, bolt axial force increased rapidly during stable failure, surged near peak load, and reached the highest force level among the tested positions.

The axial-force histories indicate staged bolt engagement: a slow, near-linear increase during the elastic stage, accelerated growth approaching the peak, and fluctuating surges in the post-peak regime. The pronounced post-peak fluctuations likely reflect dynamic energy exchange and dissipation between the bolt and the progressively fractured rock mass. When crack growth induces local dilation, the bolt provides immediate confinement through transient increases in axial force; energy is subsequently dissipated through small-scale slip and/or additional fragmentation, which contributes to a more stable failure evolution. Therefore, bolt axial force was not merely a passive response to loading, but a key mechanistic variable that restrained crack development and regulated damage progression.

**Fig 19:**
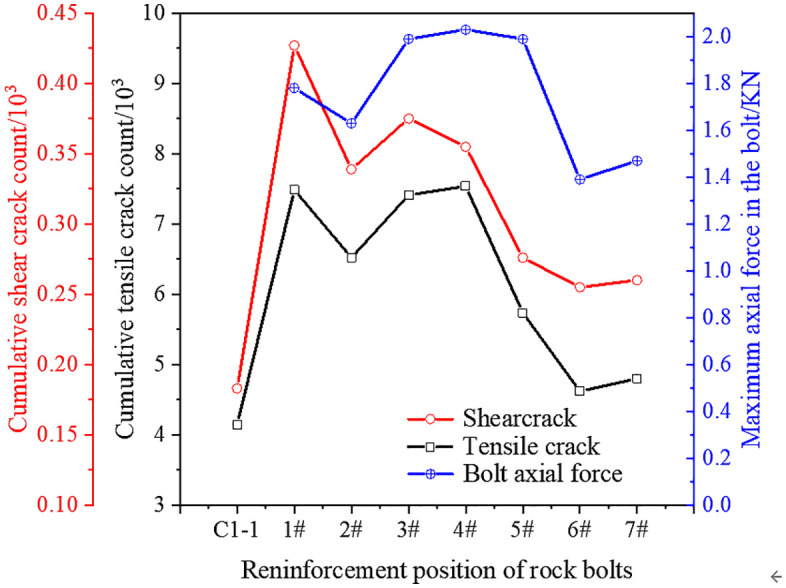
Total numbers of tensile and shear cracks at complete failure of the specimens and the corresponding maximum bolt axial force.

Building on Fig 18, Fig 19 compares the final counts of tensile and shear cracks at complete failure and the corresponding maximum bolt axial forces across the eight schemes.

First, total crack number varies nonlinearly with anchoring position. The unanchored and distantly anchored specimens (positions 6# and 7#) exhibit the fewest cracks, primarily because their lower stiffness leads to failure at relatively small loads rather than because cracking is effectively suppressed. In contrast, positions 1# and 2# show substantially more cracking, consistent with strength degradation and rapid crack propagation. Specimens within the optimal range (positions 3# to 5#) can show higher crack counts because they sustain larger loads and dissipate more energy before failure; nevertheless, they exhibit delayed crack propagation and a more stable failure process.

The spatial pattern of maximum bolt axial force closely mirrors the observed crack suppression effectiveness. Axial force level reflects the degree of bolt constraint: distant positions (6# and 7#) exhibit the lowest axial force with minimal confinement and nearly unrestrained crack development; positions 1# and 2# develop moderate axial force that engages deformation but does not fully control the primary crack path; by contrast, axial force peaks at the optimal positions (3# to 5#). These results indicate that high bolt axial force provides strong radial confinement, increases local stiffness and crack resistance near the hole, delays crack coalescence, and improves post-peak residual strength.

Therefore, maximum bolt axial force can serve as a quantitative indicator of crack inhibition by anchorage, reflecting both the bolt stress state and, indirectly, the degree of rock damage and reinforcement synergy. In essence, anchorage optimization seeks to couple crack path control, stiffness enhancement, and effective mobilization of bolt axial force.

### Energy evolution analysis

From an energy-transformation perspective, deformation and failure of rock under external loading is fundamentally a process of energy exchange. The typical stages of deformation—crack closure, elastic deformation, crack initiation and propagation, plastic deformation, and unstable failure—correspond to energy input, storage, dissipation, and release. During loading, the macroscopic mechanical response evolves concurrently with the conversion among multiple energy components, including elastic strain energy, fracture surface energy, plastic dissipation, radiated energy, and kinetic energy [56,57].

In the PFC model, the primary energy components include boundary work, bond energy, kinetic energy, frictional energy, and strain energy. Boundary work represents the work performed by boundary forces (external loading) on the particle assembly and corresponds to the total input energy, (*U*). Strain energy is the work associated with overcoming interparticle contact forces during loading. The boundary work accumulated through the upper and lower loading plates and the lateral walls is treated as the elastic energy, (*U*^e^). Bond energy represents the energy consumed by deformation and rupture of interparticle bonds. Frictional energy represents the energy dissipated by frictional sliding at contacts. Kinetic energy is the sum of the kinetic energy of all particles. The dissipated energy, (*U*^d^), is defined as the sum of bond energy, frictional energy, and kinetic energy. Accordingly, the total input energy equals the sum of elastic energy and dissipated energy:


$$W=U=U^{e}+U^{d}$$ (9)

Where *U* is the total energy (J·cm ⁻ ³), *U*^d^ is the dissipated energy (J·cm ⁻ ³), and *U*^e^ is the elastic energy (J·cm ⁻ ³). Under uniaxial compression without confining pressure (*σ*_2_ = *σ*_3_ = 0), t the energy terms are calculated as:


$$\text{U}=\int_{\text{0}}^{\epsilon_{\text{1}}}\sigma_{\text{1}}\text{d}\epsilon_{\text{1}}$$ (10)

$$U^{e}=\frac{\text{1}}{\text{2}}\sigma_{\text{1}}\epsilon_{\text{1}}^{e}=\frac{{\sigma_{\text{1}}}^{\text{2}}}{\text{2}E}$$ (11)

Where *E* is the unloading *E* in the linear-elastic stage (GPa), and ε is the specimen strain. Therefore, based on Eqs 10 and 11, the dissipated energy is computed as:


$$U^{d}=U-U^{e}$$ (12)

**Fig 20:**
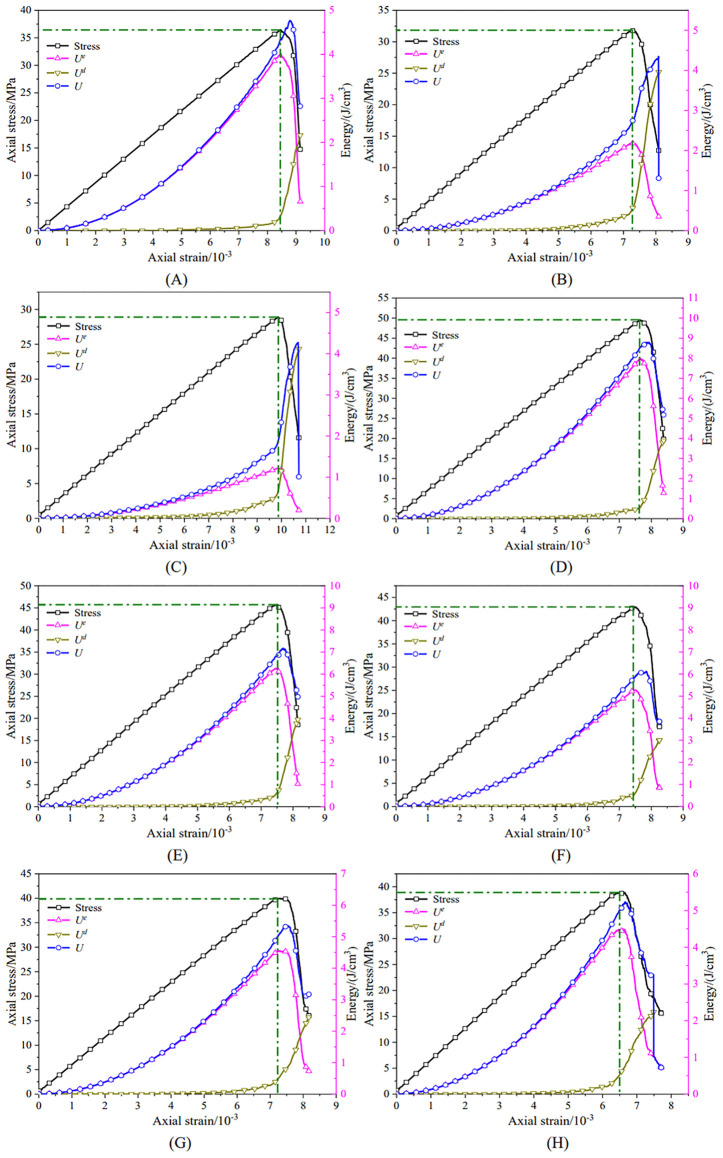
Energy evolution trends during uniaxial compression of the specimens. (A) unanchored specimen with a hole defect;(B) position 1#;(C) position 2#;(D) position 3#;(E) position 4#;(F) position 5#;(G) position 6#;(H) position 7#.

Based on these definitions, Fig 20 shows the energy evolution curves for the unanchored specimen and specimens with different anchorage positions during uniaxial compression. The curves provide a mechanistic view of deformation and fracture development and visually reflect the progressive instability process.

At peak strength, the total input energy (*U*) exhibited a systematic dependence on anchorage position. The 2# specimen had the smallest (*U*) (4.26 J/cm³; Fig 20C), followed by the 1# specimen (4.34 J/cm³; Fig 20B), whereas the 3# specimen reached the maximum (8.80 J/cm³; Fig 20D), which is approximately 1.85 times that of the unanchored defective specimen (Fig 20A). As the anchorage position moved farther from the hole center, (*U*) decreased and approached a stable level by position 7# (5.23 J/cm³; Fig 20H). The elastic energy (*U*^e^) followed a similar trend to (*U*).

In contrast, the dissipated energy (*U*^d^) at peak strength showed a different pattern. Specimens at positions 2# and 1# exhibited slightly higher (*U*^d^) than the other cases, with position 2# attaining the maximum value (4.10 J/cm³). At peak strength, the energy budget was dominated by elastic energy, with dissipated energy accounting for a smaller fraction. After the onset of unstable failure, the energy partition shifted, with dissipated energy becoming dominant and elastic energy becoming secondary. This transition indicates that the damage accumulation and instability of anchored defective specimens were closely associated with energy dissipation, and that anchorage position substantially altered the energy evolution pathway of red sandstone. Accordingly, energy-based indicators may provide useful references for the early warning of instability-related engineering hazards.

Strength failure of rock masses under high-stress conditions does not necessarily lead to global instability. Conventional strength theories and failure criteria have limited ability to explain the variability of rock strength and the evolution of overall instability during loading. From the onset of energy accumulation to subsequent energy release, the system must pass through a state characterized by a maximum elastic energy, as implied by the intrinsic linkage between these stages.

The elastic energy (*U*^e^) reflects the capacity of the rock to store energy. As *U*^*e*^ increases, greater external work is required to drive the rock to failure. Therefore, $U_{max}^{e}$ can be used to characterize the energy-storage capacity and relative failure difficulty of different rocks, or of the same rock under different stress states. In this study, $U_{max}^{e}$ obtained from numerical simulations was defined as the upper limit of elastic energy storage density. The ratio of this storage limit to the elastic energy, *K*_max_ was defined as the maximum elastic energy conversion rate, as given in Eq 13:


$$K_{max}=\frac{U_{max}^{e}}{U^{e}}$$ (13)

**Fig 21:**
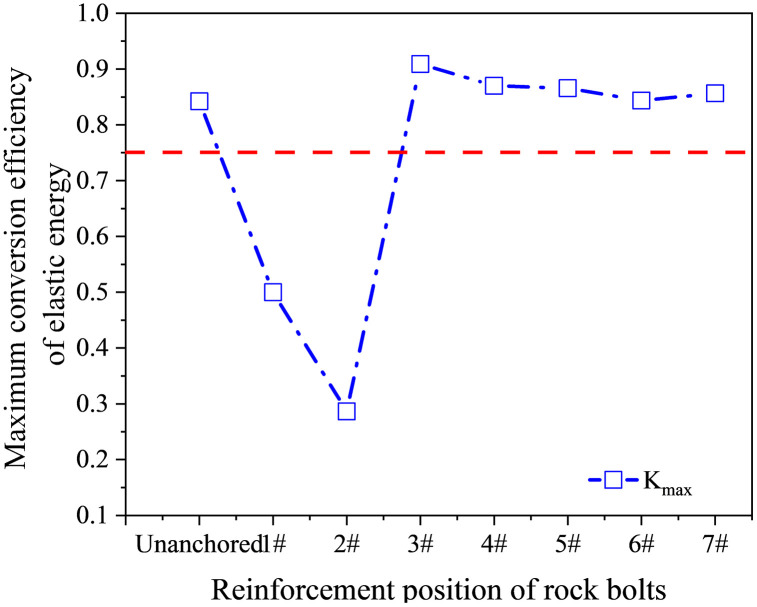
Trend of the maximum elastic energy conversion rate for the specimens.

The maximum elastic energy conversion rates under different conditions are shown in Fig 21, where the red dashed line denotes the mean value (0.75) for the corresponding set of conditions. For defective specimens anchored at positions 1# and 2# under uniaxial compression, *K*_max_ remained comparatively low. The 2# specimen exhibited the smallest *K*_max_ (0.29), indicating the weakest energy-storage capacity and the highest propensity for failure under this configuration. In contrast, the 3# specimen achieved the largest *K*_max_ (0.91), reflecting the strongest energy-storage capacity and the greatest resistance to failure. As the anchorage position moved farther from the hole center, *K*_max_ decreased gradually and eventually stabilized at approximately 0.85, which was slightly higher than that of the unanchored specimen under uniaxial compression.

### Analysis of displacement field at specimen failure

**Fig 22:**
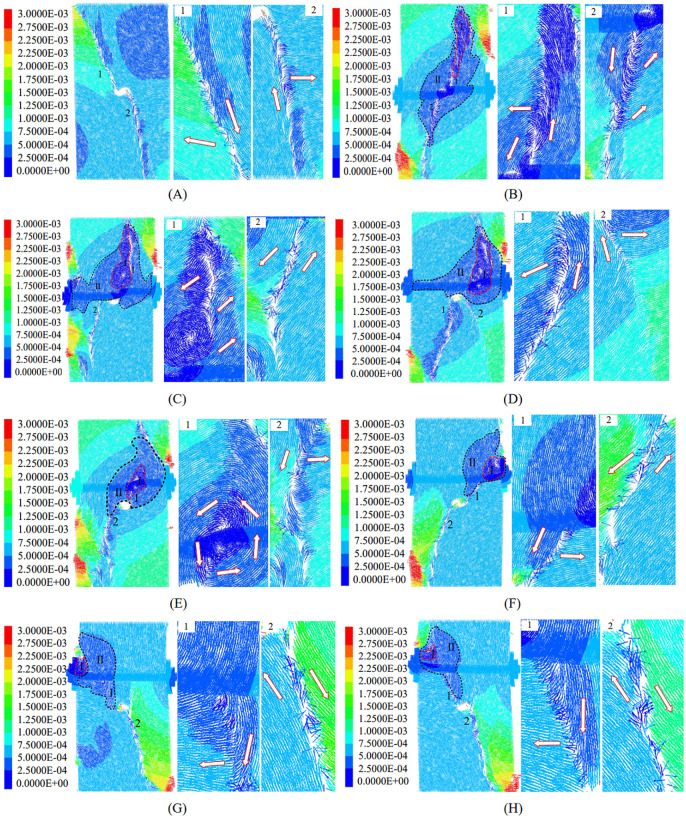
Particle displacement vector distribution at complete failure of the specimen. (A) unanchored speciment;(B) position 1#;(C) position 2#;(D) position 3#;(E) position 4#;(F) position 5#;(G) position 6#;(H) position 7#.

To further elucidate the failure characteristics associated with different anchorage configurations, the particle displacement vector field at the instant of specimen failure was extracted and plotted (Fig 22). In Fig 22, the arrows denote the displacement direction of particles. By comparing the displacement vectors on opposite sides of macroscopic cracks, the dominant failure mode can be inferred. Two representative fracture zones (labeled 1 and 2) are enlarged for detailed inspection.

For the unanchored specimen (Fig 22A), particles on opposite sides of macroscopic cracks 1 and 2 moved in opposite directions, indicating a tensile–shear mixed failure mode.

The failure mode and damage distribution of anchored specimens varied with anchorage position. At position 1# (Fig 22B), the crack morphology and particle motion were similar to those of the unanchored specimen, again reflecting a tensile–shear mixed failure. Based on displacement magnitude, the stable zone (Zone I; near-zero displacement) was narrow and elongated, whereas the damage zone (Zone II; small displacement) was distributed approximately symmetrically above and below the anchored region. At position 2# (Fig 22C), a clearer differentiation in failure mode was observed: the upper crack (crack 1) was dominated by tensile opening, whereas the lower crack (crack 2) was governed primarily by shear. The stable zone formed a concentric displacement field around the bolt with an overall counterclockwise rotation. The damage zone was concentrated above the cavity and exhibited an approximately triangular distribution.

At position 3# (Fig 22D), the specimen exhibited the best local integrity, with no pronounced macroscopic crack developing above the anchored region. The dominant cracks (cracks 1 and 2) were located at the lower-left and lower-right sides of the cavity and showed tensile–shear mixed characteristics. The stable zone was markedly enlarged, while the damage zone displayed an overall right-handed rotational tendency and an asymmetric deformation pattern. At position 4# (Fig 22E), a counterclockwise-rotating, quasi-circular displacement field developed around the bolt in the upper part of the cavity, where only microcracking occurred. The macroscopic crack at the lower-left side of the cavity (crack 2) remained tensile–shear mixed. In this configuration, both the stable and damage zones were reduced in extent.

The displacement-field patterns for positions 5#, 6#, and 7# (Figs 22F–H) were similar. No pronounced cracking was observed above the bolt. Crack 1 (between the cavity and the bolt) exhibited tensile–shear mixed behavior, whereas crack 2 (below the cavity) was predominantly shear. For these positions, the stable and damage zones were further reduced, and the zones for positions 6# and 7# converged to similar sizes, suggesting comparable restraint effects.

Overall, analysis of the displacement vector fields at failure demonstrates how anchorage position governs the failure behavior of cavity-containing sandstone and the effectiveness of crack-growth inhibition. Bolt installation substantially altered the displacement trajectories and movement patterns of particles around the cavity. At effective anchorage positions (e.g., 3# and 4#), the bolt promoted the formation of a localized or concentric displacement field centered on the bolt. This mechanism redirected and confined deformation that would otherwise localize toward free surfaces into the vicinity of the bolt, thereby partitioning large-scale continuous deformation into multiple localized, discontinuous deformation domains. Consequently, the development of through-going macroscopic cracks was effectively suppressed.

## Conclusions

This study investigated how bolt position controls the mechanical response and failure mechanisms of red sandstone specimens containing a circular cavity by combining laboratory tests with PFC2D simulations. The main conclusions are as follows:

(1) The laboratory tests confirmed that bolt position strongly influenced mechanical properties and failure patterns. When the bolt intersected the cavity or was placed adjacent to the cavity boundary (positions 1# and 2#), the specimens exhibited marked reductions in strength and stiffness, with pre-peak stress drops and predominantly brittle failure. Peak strength, *E*, and crack initiation stress decreased substantially. In contrast, when the bolt was installed at a moderate offset outside the cavity (positions 3# and 4#), both strength and stiffness increased significantly, and the post-peak response shifted from brittle to a more progressive, ductile-like behavior. The above mechanical indicators increased by more than 40%. Bolt position also governed the crack initiation pathway and the final failure morphology. Anchorage near the cavity promoted stress concentration at the cavity roof and produced a V-shaped collapse. Anchorage at an intermediate offset led to partitioned tensile–shear failure zones, whereas anchorage farther from the cavity increased the overall damage extent and resulted in more complex fracture morphologies.
(2) The numerical simulations reproduced the mechanical response and crack evolution of anchored, cavity-containing sandstone under uniaxial compression. The PFC^2D^ results indicated that bolt position exerted a dominant influence on mechanical performance and crack suppression, with a clear optimal anchorage range (positions 3#–5#). Within this range, peak strength and *E* were simultaneously maximized and crack propagation was effectively restrained. In this configuration, the bolt was fully mobilized, substantially limiting deformation and reducing the damage extent through the formation of a localized confinement zone. In contrast, bolts placed either too close to or too far from the cavity provided limited reinforcement. Specimens with the optimal bolt position (e.g., 3#) exhibited the strongest energy-storage capacity (maximum elastic energy conversion rate, (*K*ₘₐₓ = 0.91) and the greatest resistance to failure, whereas specimens anchored near the cavity showed the weakest energy-storage capacity (*K*_min_ = 0.29) and the highest susceptibility to instability. As the anchorage position moved farther from the cavity, energy-storage capacity decreased gradually and then stabilized. Moreover, the failure process transitioned from an elastic-energy-dominated state to a dissipation-dominated state, indicating that energy evolution metrics can effectively characterize damage development in rock.
(3) These findings provide a quantitative basis for anchorage optimization and stability assessment in engineering applications. An optimal anchorage interval was identified (12–18 mm outside the cavity in this study), within which load-bearing capacity, stiffness, and crack resistance were maximized. Anchorage too close to the cavity should be avoided because it can degrade mechanical performance. Energy-based indices, such as the elastic energy conversion rate, may serve as candidate criteria for rock-mass stability evaluation and early-warning applications, thereby providing theoretical support for support design and risk mitigation.

This study was conducted under uniaxial loading and considered only one lithology (red sandstone), a fixed cavity size, and a single bolt diameter, while keeping all anchorage parameters except position constant. The effects of more complex conditions—such as triaxial stress states, confining pressure, anisotropy, and realistic rock-mass discontinuity structures—were not addressed. In addition, the crack-restraint mechanism analysis adopted simplifying assumptions regarding interactions such as shear behavior, bond response, and bolt–rock interface contact, to isolate the effect of bolt position. Although these simplifications facilitate mechanistic interpretation, they differ from the coupled, multi-factor behavior of anchorage systems in field settings and may limit direct extrapolation to engineering practice. Future work should consider more realistic stress environments, additional lithologies, multi-scale anchorage parameters, and complex loading paths to systematically characterize bolt anchorage mechanisms under representative geological and mechanical conditions.

## Supporting information

S1 File Minimal data set. (XLSX)
